# Herpes encephalitis presenting as mild aphasia: Case report

**DOI:** 10.1186/1471-2296-7-22

**Published:** 2006-03-24

**Authors:** Omar A Khan, Allan Ramsay

**Affiliations:** 1 Department of Family Medicine, University of Vermont, Burlington, Vermont 05405, USA

## Abstract

**Background:**

Encephalitis presenting as a change in mental status can be challenging to recognize in the primary care setting. However, early detection via a low threshold of suspicion can be useful, leading in turn to early treatment and improved survival.

**Case presentation:**

We present a case which we consider relevant to primary care practitioners. The patient in question presented with relatively mild mental status changes, progressing to confusion, dysnomia and delirium over a period of three days. While infection did not appear to be the leading cause on her differential diagnosis, she was found on extensive workup to have encephalitis caused by Herpes Simplex Virus type 1.

**Conclusion:**

The case is instructive for general practitioners and other clinicians to maintain vigilance for central nervous system (CNS) infections which may present atypically.

## Background

Encephalitis presenting as a change in mental status can be challenging to recognize in the primary care setting and in general inpatient medicine. Among the causes of viral encephalitis is Herpes Simplex virus type 1 (HSV-1). A low threshold of suspicion can lead to timely diagnostic testing, detection, and treatment. This in turn leads to improved survival. This case of acute mental status changes can illustrate some of these principles for the benefit of family physicians and other primary care providers.

## Case presentation

M.J. is a 59 year old woman who presented to the emergency room at a tertiary care center with a three day history of mental status changes. According to her accompanying spouse, three days prior she had eaten out and had one episode of diarrhea. Since then she had slowly become increasingly 'confused', as she described it. She had trouble finding the right words and had been using incorrect words in sentences. M.J. seemed untroubled by this change. These symptoms were accompanied by mild anorexia.

M.J. had a no significant previous medical history. She had no recent illnesses. She had experienced two vaginal births, and in true family medicine fashion, one of the medical team taking care of the patient had delivered her grandson about one year ago.

The patient emigrated from Germany thirty years ago, and English was her second language. She resided in a medium-sized northern New England town with her husband. She operated a daycare out of her home, with approximately five pre-school aged children, none of whom had recently had any serious illnesses.

On initial presentation to the Emergency Department, M.J. had no elevation in temperature. She had a mild frontal headache. Her vital signs were otherwise within normal limits. Her physical exam showed her to be alert and oriented, but with mild dysnomia on questioning. She did not have meningeal signs, but appeared mildly dehydrated. An initial computed tomography scan of the head (with and without contrast) was unremarkable for mass or bleed. However, it showed a moderate left-sided sphenoid sinusitis. Her leukocyte count, electrolytes, liver transaminases, folate, B12 and thyroid stimulating hormone were within normal limits.

She was admitted with intravenous fluids and intravenous ceftriaxone as empiric antibiotic coverage. An otolaryngology consult was obtained to determine whether the mental status changes could be attributed to the sinus findings. Throughout the next day, the patient became increasingly aphasic. Magnetic resonance imaging (MRI) of the brain was ordered and a lumbar puncture was performed. The fluid showed a leukocyte count of 75 and an erythrocyte count of 5, with a lymphocytic predominance (75%). The MRI showed left temporal lobe enhancement. The cerebrospinal fluid (CSF) was sent for polymerase chain reaction (PCR) analysis, as well as for viral cultures. While this was pending, the patient was empirically started on intravenous acyclovir for presumed herpes encephalitis.

The patient required transfer to the intensive care unit soon thereafter for airway protection, where she continued her antiviral medication. PCR testing of the spinal fluid confirmed the presence of *Herpes simplex *virus type 1 (HSV-1). M.J. spent three weeks in acute rehabilitation after her hospitalization. She was treated with intravenous acyclovir for four weeks and oral valacyclovir for an additional three weeks. M.J. has required longer term speech therapy; however, she has returned to fully independent living.

In our patient's case, the diagnosis of HSV encephalitis was first raised by the cerebrospinal fluid findings, strongly indicated by the information added by the MRI, and confirmed by the PCR testing. HSV encephalitis is an uncommon illness, with about 2 cases per 250,000 per year [1,2]. Most are caused by HSV-1, with 10% having HSV-2 as the etiologic factor. An MRI is abnormal in the majority of PCR-positive cases [3]. PCR itself has a reported sensitivity of 98% and a specificity of 94% for the detection of HSV in the CSF [4]. In general, the treatment for HSV encephalitis is to initially treat with intravenous acyclovir for 21 days [5,6], with a further period of oral antiviral medication as clinically indicated. Untreated, this particular encephalitis has a high case-fatality rate, of around 70% [7]. Even if it is treated in a timely fashion, the prognosis is only fair: approximately 40–50% would be expected to make full neurological recovery [7,8].

## Conclusion

This was a fortunate patient, who, despite a slight delay in initial diagnosis, has recovered nearly completely. It is instructive to maintain vigilance for infectious encephalitis when a patient presents with mental status changes in the primary care or hospital setting. Early recognition can prevent serious morbidity.

## Competing interests

The author(s) declare that they have no financial or non-financial competing interests.

## Authors' contributions

These authors contributed equally to this work.

## Pre-publication history

The pre-publication history for this paper can be accessed here:


